# Comparative Study of Leech-Derived Tryptase Inhibitor Genes in Four Medicinal Leech Species

**DOI:** 10.3390/biology14091247

**Published:** 2025-09-11

**Authors:** Mingkang Xiao, Fang Zhao, Tianyu Ye, Rui Ai, Lizhou Tang, Zichao Liu, Qingqian Zeng, Zuhao Huang, Gonghua Lin

**Affiliations:** 1 Key Laboratory of Jiangxi Province for Biological Invasion and Biosecurity, School of Life Sciences, Jinggangshan University, Ji’an 343009, China; xmk18379773872@163.com (M.X.); zf_lgh@163.com (F.Z.); yetianyu0220@163.com (T.Y.); 18008109213@163.com (R.A.); 2 College of Life Sciences, Jiangxi Normal University, Nanchang 330022, China; tanglizhou@163.com; 3 School of Agronomy and Life Sciences, Kunming University, Kunming 650214, China; abclzc@aliyun.com; 4 Guangdong Institute of Traditional Chinese Medicine, Guangzhou 510630, China; zqqlisa@163.com

**Keywords:** medicinal leeches, antithrombotic gene, genetic variation, eukaryotic expression, anticoagulant activity

## Abstract

Leeches are aquatic annelids of significant medicinal value, known to produce a variety of antithrombotic proteins. However, the extent of interspecies variation in these bioactive components remains poorly understood. In this study, next-generation sequencing and the *Pichia pastoris* eukaryotic expression system were employed to systematically analyze the diversity of leech-derived tryptase inhibitor (LDTI) genes and their functional activities across four dominant medicinal leech species: *Hirudinaria manillensis*, *Hirudo nipponia*, *Whitmania pigra*, and *Whitmania laevis*. Our results revealed a slightly higher genetic variation in LDTI genes and their protein among non-hematophagous leeches (*W. pigra* and *W. laevis*) compared to hematophagous species (*H. manillensis* and *H. nipponia*). Selection analysis indicated purifying selection in *H. manillensis* (*ω* = 0.47), while the other species exhibited either neutral or weak positive selection (*ω* ≥ 1). LDTI expression levels were significantly higher in hematophagous leeches, with peak expression observed in *H. manillensis* (transcripts per million = 2942.07 ± 1593.12). In vitro chromogenic substrate-based anticoagulant assays of recombinant proteins confirmed anticoagulation activity across all species, with *H. manillensis* displaying the most potent inhibition. These findings demonstrate the antithrombotic potential of all four species, regardless of their feeding ecology. Moreover, *H. manillensis* emerges as the optimal candidate for therapeutic development, owing to its superior LDTI sequence conservation, expression levels, and specific anticoagulant activity per unit concentration.

## Simple Summary

Leeches are aquatic annelids with significant medicinal value, known for producing various antithrombotic proteins. This study investigated the genetic diversity and functional activity of leech-derived tryptase inhibitor (LDTI) genes across four medicinal leech species—*Hirudinaria manillensis*, *Hirudo nipponia*, *Whitmania pigra*, and *Whitmania laevis*—using next-generation sequencing and the *Pichia pastoris* eukaryotic expression system. Our findings revealed greater genetic variation in LDTI genes among non-hematophagous species (*W. pigra* and *W. laevis*) compared to hematophagous species (*H. manillensis* and *H. nipponia*). We also observed significantly higher expression levels of LDTI in hematophagous leeches, with *H. manillensis* exhibiting peak expression and the strongest anticoagulant activity. This study highlights the antithrombotic potential of these leech species and identifies *H. manillensis* as the most promising candidate for the development of therapeutic anticoagulants due to its superior genetic conservation and bioactivity.

## 1. Introduction

Leeches (Hirudinea), belonging to the phylum Annelida and the class Hirudinea [1], represent a group of invertebrates of significant medicinal value. These animals are classified into two major taxonomic groups based on their feeding ecology: hematophagous (e.g., *Hirudinaria manillensis*) and non-hematophagous (e.g., *Whitmania pigra*). Hematophagous leeches feed on the blood of mammals (e.g., humans, cattle, horses), whereas non-hematophagous species consume the body fluids of mollusks, such as freshwater snails (e.g., *Bellamya* spp.) and freshwater mussels. During feeding, hematophagous leeches secrete various anticoagulant substances that facilitate sustained blood flow from host vessels [2]. Leeches have a long history in traditional medicine. During medieval and early modern European medical practices, *Hirudo medicinalis* was used for treatment. [3]. By feeding on areas of localized vascular congestion, leeches remove excess blood to restore humoral equilibrium. In China, they have historically been used as a traditional medicinal agent to enhance microcirculatory dynamics and facilitate the resolution of blood stasis. Clinically, leeches are indicated for blood stasis syndrome, which manifests as thrombosis, amenorrhea, and pain associated with stasis [4]. Interestingly, certain non-hematophagous leeches retain antithrombotic activity due to ancestral remnants of bioactive compounds inherited from hematophagous ancestors. For example, *W. pigra*, which preys on freshwater snails, has been repeatedly shown to possess significant anticoagulant and thrombolytic activities [5]. This pharmacological profile likely results from its close phylogenetic relationship with hematophagous Hirudo species [6], from which it inherited substantial antithrombotic compounds derived from sanguivorous ancestors.

The antithrombotic compounds in leeches are primarily peptide-based molecules, encoded by specific antithrombotic genes, the majority of which belong to multigene families [7]. Using the Asian buffalo leech (*H. manillensis*) as an example, a total of 21 antithrombotic gene families, comprising 72 distinct genes, have been identified both domestically and internationally [8]. These include 14 coagulation inhibitors, 3 platelet aggregation inhibitors, 3 fibrinolysis enhancers, and 1 tissue penetration enhancer. Another representative hematophagous leech, *Hirudo nipponia*, has been found to possess 22 antithrombotic gene families, totaling 86 antithrombotic genes [9]. Surprisingly, the non-hematophagous leech *W. pigra* also possesses a similar number and composition of antithrombotic genes to *H. manillensis*. Within its 21 antithrombotic gene families, a total of 79 antithrombotic genes were identified. Furthermore, studies indicate that *Whitmania laevis*, a monophyletic branch of *W. pigra*, also exhibits 21 antithrombotic gene families, totaling 76 antithrombotic genes [10,11]. Among the numerous antithrombotic genes in leeches, hirudin—currently the most potent natural thrombin inhibitor known—has been extensively studied [12]. In contrast, research on other antithrombotic gene families has been considerably less extensive. LDTI (leech-derived trypsin inhibitor), a Kazal-type serine protease inhibitor of leech origin, is the first small molecule known to bind tightly to and specifically inhibit human trypsin activity in the nanomolar range [13]. This small molecule, composed of 46 amino acid residues, was initially isolated from *H. medicinalis* and has a molecular weight of 4378 Da [14]. It contains three disulfide bonds and exerts its specific inhibitory effect by tightly binding to two active sites within the tetrameric structure of trypsin [15]. Notably, the LDTI gene belongs to a single-copy gene family. In the majority of leech species—including the hematophagous *H. manillensis* and *H. medicinalis*, as well as the non-hematophagous *W. pigra* and *W. laevis*—the LDTI gene family consists of only a single gene copy. An exception is *Whitmania acranulata*, which possesses six LDTI genes generated through recent tandem duplication. This single-copy nature makes LDTI an ideal model for investigating interspecies variation [11].

Current research on antithrombotic genes primarily focuses on *H. medicinalis*, with other species receiving considerably less attention. Asia is a major reservoir of medicinal leech resources [16,17]. In China alone, nearly 100 leech species have been documented. Our preliminary studies have revealed that beyond the previously characterized *H. manillensis*, *H. nipponia*, *W. pigra*, and *W. laevis*, additional species such as *W. acranulata* [11] and *Hirudo tianjinensis* [9] possess a rich repertoire of antithrombotic genes. It is noteworthy that annelids, exemplified by leeches, exhibit significant genomic variability, driving extensive diversification within gene families and leading to dynamic evolutionary events, including lineage-specific gene birth-and-death processes [18,19]. Moreover, even when orthologous antithrombotic genes are present across species, functional degeneration or pseudogenization may occur. As a result, not all paralogs maintain antithrombotic activity. For instance, while *H. manillensis* and *W. pigra* possess five and seven hirudin genes, respectively, functional validation studies confirm that three exhibit anticoagulant activity in *H. manillensis* [8], while only one retains functionality in *W. pigra* [11]. The inactive variants are collectively classified as hirudin-like analogs. Moreover, significant variations in hirudin gene expression levels have been documented both across species [20,21] and within individual species [6]. This phenomenon underscores that antithrombotic genes may exhibit considerable inter- and intraspecific divergence across three key dimensions: sequence variation, transcriptional regulation, and functional activity. Therefore, adopting a multi-species comparative approach is crucial for fully elucidating the function of these genes and their protein products.

This study focuses on four leech species with notable medicinal value and evolutionary significance: *H. manillensis*, *H. nipponia*, *W. pigra*, and *W. laevis*. *H. manillensis*, the type species of Hirudinidae family, is widely distributed across Southeast Asia [22,23]. In China, it holds particular significance as a medicinal material, having been formally monographed in the Yunnan Provincial Standards for Chinese Materia Medica (Standard Code: YunYPBZ-0199-2013) [24]. *H. nipponia* is currently the only sanguivorous leech species monographed in the Pharmacopoeia of the People’s Republic of China, conferring substantial value for antithrombotic drug development. In contrast, *W. pigra* is also included in the pharmacopoeia [25]. Its non-hematophagous nature has made this species a subject of ongoing pharmacological debate [26]. The primary objective of this study is to elucidate the genetic and functional characteristics of LDTI genes in four leech species—*H. manillensis*, *H. nipponia*, *W. pigra*, and *W. laevis*—through a multidimensional analysis of sequence variations, transcriptional dynamics, and functional activities. This integrated approach aims to facilitate the development of novel antithrombotic proteins while uncovering evolutionary trajectories associated with hematophagous behavior.

## 2. Materials and Methods

### 2.1. DNA and RNA Sequencing

Live specimens of *H. manillensis*, *H. nipponia*, *W. pigra*, and *W. laevis* were collected from the ditches next to the paddy fields in various locations across China. Species identification was conducted following using the authoritative taxonomic keys and descriptions provided in Fauna Sinica: Annelida, Hirudinea [1]. Key diagnostic characteristics included total body length and width, body shape, coloration, annulation patterns, and position of the genital pore. The specific collection details for each species were as follows: *H. manillensis* from Honghe, Yunnan (YNHH, 102.58° E, 23.31° N); *H. nipponia* from Baodi, Tianjin (TJBD, 117.48° E, 39.47° N); *W. pigra* from Yibin, Sichuan (SCYB, 105.31° E, 28.18° N); and *W. laevis* from Qijiang, Chongqing (CQQJ, 106.64° E, 29.03° N). For each sampling site, ten leeches were randomly selected for dissection. After removal of the digestive tract, cephalic tissues were excised and processed for total DNA extraction using the DNeasy Blood & Tissue Kit (QIAGEN, Hilden, Germany), with DNA isolated individually for each specimen. The quality and integrity of the extracted DNA samples were assessed using NanoDrop spectrophotometry (NanoDrop Technologies, Wilmington, DE, USA) and agarose gel electrophoresis (1% gel, 120 V, 30 min). Concurrently, total RNA was extracted from cephalic tissues using TRIzol^TM^ RNA Isolation Reagent (Thermo Fisher Scientific Inc., Waltham, MA, USA), followed by purification with the RNeasy Mini Kit (Qiagen, Chatsworth, CA, USA) and on-column DNase I digestion. DNA and RNA extracts that passed quality control were used to construct libraries with ~350 bp insert sizes using Illumina^®^-compatible reagents. Sequencing was conducted on the BGISeq-500 platform (BGI Genomics, Shenzhen, China) with (i) whole-genome resequencing using 150 bp paired-end reads and (ii) strand-specific RNA sequencing (RNA-Seq) using 150 bp paired-end reads. Raw sequencing reads were processed using Fastp v0.20.0 [27] to trim adapters and remove low-quality bases, generating high-quality clean reads for each sample, which were then used for downstream bioinformatic analyses.

### 2.2. Sequence Extraction and Genetic Variation Analysis

The initial de novo assembly of the genome-resequenced clean reads was performed with Megahit v1.2.9 [28], producing the corresponding genome contig sequence file for each sample. Next, de novo transcriptome assembly of the RNA-seq clean reads was performed with Trinity v2.9.0 [29], generating the unigene sequence file for each sample. A previously published LDTI gene sequence (Supplementary File S1) [13] was used as bait to screen for homologous sequences from the unigene files with BLAST v2.13.0+ [30]. Putative coding sequences were identified and extracted using the GT-AG splicing rule in combination with sequence alignment performed in MEGA v11.0.13 [31]. For genes or samples with low expression levels, where the unigene files failed to yield complete coding sequence regions, the following approach was employed: each exon, flanked by approximately 50 bp of its upstream and downstream sequence, was used as the bait. Homologous sequences were then screened from the genome contig files using BLAST. These sequences were aligned with MEGA, and the corresponding exon regions were excised.

A systematic analysis of the coding sequence regions was performed for each gene. Sequence files were saved in FASTA format, and codon-based alignment was conducted using the ‘Align by Muscle (Codons)’ function in MEGA software. Concurrently, DnaSP v6.12.03 [32] was used to calculate the number of Variable Sites (VS) and Haplotype Number (HN). Watterson’s Theta Diversity was calculated using DAMBE v7.3.5 [33]. Next, the nucleotide sequences were translated into their corresponding amino acid sequences. These sequences were analyzed using DAMBE software to determine the number of variable sites, haplotype number, and Watterson’s Theta at the amino acid level.

### 2.3. Phylogenetic and Molecular Evolutionary Analysis

A phylogenetic tree is a dendrogram that depicts evolutionary relationships among biological taxa through branching topology, facilitating the inference of phylogenetic affinities and divergence histories [34]. Given the complex evolutionary dynamics within the leech LDTI gene family, we performed comprehensive phylogenetic reconstruction with an expanded taxonomic sampling to reduce stochastic errors and improve topological reliability, thereby more accurately resolving interspecific evolutionary patterns. To this end, LDTI genes from *H. tianjinensis*, *H. medicinalis*, and *W. acranulata* were included as references. Multiple sequence alignment of LDTI genes from seven leech species was conducted using MEGA, followed by maximum likelihood phylogenetic tree construction with IQ-TREE v1.6.12 [35].

This study systematically analyzed selective pressures on LDTI genes by calculating the nonsynonymous-to-synonymous substitution rate ratio (*ω* = *dN/dS*) using the site models, branch model, and branch-site model in the CodeML module of the PAML-X v1.2 package [36]. The Site Model (Models 0, 2, 7, and 8) was primarily used to analyze genome-wide selective pressures on LDTI genes and identify potential positively selected sites. Models 0 (one-ratio model) and 2 (positive selection model) classified codon sites into three selective pressure categories: purifying selection (*ω* < 1), neutral selection (*ω* = 1), and positive selection (*ω* > 1). Model 7 (beta model) divides codon sites into 10 discrete categories under purifying selection, whereas Model 8 (*beta* + *ω*) extends this framework by incorporating an additional class for positively selected sites (*ω* > 1). The branch model was subsequently applied to assess lineage-specific selective pressures across different clades. Finally, the branch-site model was utilized to detect episodic diversifying selection acting on specific evolutionary branches of LDTI genes. To enhance the accuracy of *ω*-value estimates and minimize stochastic errors, we incorporated LDTI gene sequences from *H. medicinalis*, *H. tianjinensis*, and *W. acranulata* as background branches. Each study species was sequentially assigned as the foreground branch, with the remaining taxa as background, enabling the detection of lineage-specific selection patterns through branch-site model *ω*-value analysis. This comprehensive approach revealed distinct selective pressure types and adaptive evolutionary mechanisms acting on LDTI genes across leech species.

### 2.4. Gene Expression Analysis

In this study, all coding sequences derived from the whole-genome structural annotation were used as templates. Sequence indexes were generated using Salmon v1.0.0 software [37], and transcriptomic reads from each sample were subsequently aligned to these index files. Using a k-mer size of 31 as the primary parameter, transcripts per million (TPM) values were calculated for each coding sequence and used as relative expression levels across all samples. Differential expression patterns of LDTI genes among the species were analyzed using SPSS v25.0 [38]. A One-Sample Kolmogorov–Smirnov test (used to evaluate whether the sample data follows a normal distribution) indicated that gene expression levels significantly deviated from a normal distribution across all species (*p* < 0.001). As a result, non-parametric statistical methods were employed for subsequent analyses. The Kruskal–Wallis test (Independent Samples Test) was first applied to assess overall expression differences among species. When significant differences were identified, pairwise comparisons were conducted using the Mann–Whitney U test (Independent Samples Test) to pinpoint specific interspecies variations in gene expression.

### 2.5. Pichia Pastoris Expression

After removing signal peptide sequences and stop codons, the coding sequences of LDTI genes from four leech species were synthesized by Shanghai Biological Engineering Co., Ltd. (Shanghai, China) These sequences were subcloned into the pPIC9K expression vector to construct recombinant plasmids for transformation into *Escherichia coli*. The recombinant *E. coli* strains were then inoculated into LB liquid medium and cultured overnight with shaking. Circular plasmid DNA was extracted using the SanPrep Column Plasmid Mini-Preps Kit (Sangon Biotech Co., Ltd., Shanghai, China) and then linearized with the SpeedyCut SacI (Takara Bio Inc., Shiga, Japan) restriction enzyme. The separation and detection were subsequently performed using agarose gel electrophoresis. The successful linearization of the circular plasmid DNA was determined based on the positional shift of the electrophoretic bands. Finally, the linearized plasmid DNA was further purified with the SanPrep Column PCR Product Purification Kit (Sangon Biotech Co., Ltd., Shanghai, China) to ensure high DNA purity.

The purified, linearized plasmid DNA was transformed into chemically competent GS115 cells via chemical transformation. The transformed cells were plated onto Yeast Extract Peptone Dextrose (YEPD) agar medium containing 0.25% dextrose and incubated for 3–5 days until yeast colonies appeared. To select for high-resistance *Pichia pastoris* clones, positive transformants were sequentially transferred to YEPD media containing increasing concentrations of Geneticin (0.5%, 1%, and 2%) for stepwise selection. High-resistance clones were inoculated into BMGY (Buffered Glycerol-complex Medium with Yeast Extract) medium for large-scale cultivation. Genomic DNA was then extracted using a yeast genomic DNA rapid extraction kit. PCR amplification was performed using the extracted DNA as the template, followed by agarose gel electrophoresis to analyze the amplification products. The successful integration of the target gene into the *P. pastoris* genome was confirmed by the presence of expected electrophoretic bands and the growth of yeast colonies.

Following the successful verification of target gene integration, yeast cultures were transferred from BMGY to BMMY (Buffered Methanol-complex Medium with Yeast Extract) medium to induce protein expression via methanol. The expressed proteins via ammonium sulfate precipitation and subsequently subjected to dialysis-mediated desalting using a standard regenerated cellulose membrane (molecular weight cut-off: 2 kDa, width: 45 mm). Prior to use, the membrane was rinsed and equilibrated by soaking in 0.1% EDTA for 1 h, followed by extensive rinsing and a 15-min immersion in deionized water. A 1.5 mL aliquot of the protein sample was then loaded into the prepared dialysis membrane and dialyzed against 1× PBS (phosphate-buffered saline) buffer under continuous agitation at 500 rpm using a magnetic stirrer for 48 h.

To sustain a constant chemical potential gradient, the external PBS buffer was replaced at 8-h intervals. Protein concentration was determined using a Quawell Q5000 microvolume spectrophotometer (Quawell Technology Inc., Sunnyvale, CA, USA). Prior to measurement, the pedestal was meticulously cleaned with lint-free wipes. A 1 µL aliquot of 1× PBS buffer was used to establish the baseline by selecting the “Protein A280” mode and performing a blank measurement. Subsequently, 1 µL of each protein sample was carefully loaded onto the lower measurement pedestal. The arm was closed, and the measurement was initiated by clicking “Measure”. To prevent cross-contamination, the sensor surfaces were rigorously cleaned with PBS and dried between each sample. The resulting concentration values were recorded for further analysis.

### 2.6. Anticoagulation Test

We employed the Sienco model coagulation and platelet function analyzer (manufactured by Viscell, Cambridge, UK) to assess the anticoagulant activity of the target protein using blood viscoelastic dynamics detection technology. Aliquots of 500 μL of porcine blood anticoagulated with 3.8% sodium citrate were prepared. After adding 100 μL of the target protein solution, 20 μL of 0.25 M calcium chloride was introduced to reverse citrate-mediated anticoagulation through calcium chelation, thereby restoring physiological coagulation competence. A 360 μL sample of the mixed blood was transferred into a measurement cup, and the instrument’s ultrasonic transducer was used to oscillate at 200 Hz, enabling real-time monitoring of viscoelastic impedance changes during coagulation. These measurements were subsequently converted into a hemostatic signature curve, reflecting the dynamics of clot formation.

We evaluated recombinant LDTI proteins from four leech species as the study targets, conducting three independent experimental replicates per group. Key parameters monitored included (1) Activated Clotting Time (ACT), with a reference range of 100–240 s, and values >240 s indicating clinically significant prolongation; (2) Clot Rate (CR), with a normal interval of 10–35, where values <10 indicate pathological hypocoagulability; and (3) Platelet Function (PF), with a physiological threshold >1, and values ≤1 correlating with substantially elevated hemorrhagic risk. Through quantitative analysis of coagulation signature profiles, we assessed the anticoagulant activity of the target proteins. Typically, when an anticoagulant-active target protein is present, the addition of calcium ions results in either retarded clot formation kinetics or complete suppression of the coagulation cascade, leading to discernible divergence in hemostatic properties compared to control cohorts.

Differences in ACT values among different species were analyzed using SPSS v25.0. The normality of the ACT value distribution for all species was assessed using the one-sample Kolmogorov–Smirnov test, which indicated a significant deviation from normality (*p* < 0.05). Therefore, non-parametric statistical methods were employed. First, the Kruskal–Wallis H test for k independent samples was applied to evaluate overall differences in ACT values across species. When a significant difference was detected, post hoc pairwise comparisons were conducted using the Mann–Whitney U test, thereby identifying which specific pairs of species differed significantly.

## 3. Results

### 3.1. Genetic Variant Profiling

Complete LDTI coding sequences were successfully identified for all ten individuals of each species: *H. manillensis*, *H. nipponia*, *W. pigra*, and *W. laevis* (Supplementary File S2). Our analysis revealed significant interspecific differences in genetic diversity (Table 1). At the DNA level, a total of 21 variant sites and 22 haplotypes were identified across the four species. *H. manillensis* exhibited the fewest variant sites and haplotypes, while *W. laevis* had the most variant sites, and *W. pigra* showed the highest haplotype diversity. At the protein level, 7 variant sites and 10 haplotypes were identified. *W. pigra* displayed the highest counts for both parameters (4 variant sites and 4 haplotypes), significantly exceeding those of the other species. Watterson’s Theta analysis indicated that genetic diversity in *W. laevis* (at the DNA level) and *W. pigra* (at the protein level) was significantly higher than in the other species, while no significant differences were found among the remaining taxa.

### 3.2. Phylogenetic and Selection Pressure Analysis

**Figure 1:**
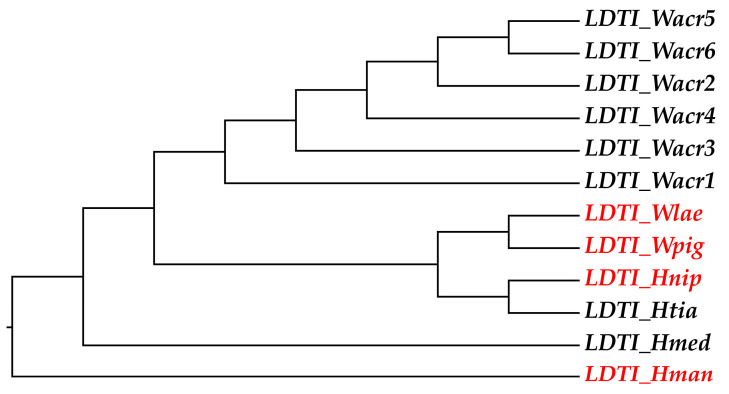
Species tree constructed from LDTI gene nucleotide sequences of *Hirudinaria manillensis*, *Whitmania pigra, Hirudo nipponia, Whitmania acranulata* (*LDTI_Wacr*1–6), *Hirudo tianjinensis*, *Whitmania laevis*, and *Hirudo medicinalis*. Note: The red font indicates the target sequences analyzed in this study.

Phylogenetic analysis of the LDTI gene family across seven leech species (Figure 1), coupled with comprehensive selection pressure analysis, revealed a key evolutionary pattern: despite overall conservation (primarily driven by purifying selection), this gene family shows distinct positive selection signals. While the entire gene is under constraint, the Site model identified a substantial proportion of sites undergoing strong positive selection. Among these, Bayes Empirical Bayes analysis confidently identified nine key sites (posterior probability > 95%). Furthermore, the Likelihood Ratio Test provided strong support for the presence of positive selection: the comparison of Model 0 versus Model 2 yielded a statistically significant result (*p* < 0.001), strongly favoring Model 2, which included sites under positive selection. This conclusion was further supported by the Model 7 vs. Model 8 comparison (*p* < 0.001). Branch model analysis revealed distinct selection patterns: *H. manillensis* was under purifying selection, while *H. nipponia*, *W. pigra*, and *W. laevis* experienced neutral selection or weak positive selection pressure. Branch-site model analysis showed that in both *H. manillensis* and *W. laevis*, purifying and neutral selection accounted for 45% and 55% of sites, respectively, with no positively selected sites detected. In contrast, *H. nipponia* and *W. pigra* exhibited all three selection pressures (Table 2). Notably, *W. pigra* uniquely possessed two potential positively selected sites (posterior probability > 0.95), a signature not observed in any other species examined.

### 3.3. Gene Expression

Analysis of relative expression levels based on transcriptomic data revealed striking contrasts: the LDTI gene in *H. manillensis* exhibited the highest TPM value (2942.07 ± 1593.12, mean ± SD), while *W. pigra* showed the lowest expression (32.03 ± 48.57, mean ± SD), constituting a 91.8-fold difference between species. Kruskal–Wallis test analysis indicated significant differences in LDTI gene expression among at least two leech species (*p* < 0.001). The rank means revealed the highest value in *H. manillensis* (34.90), corresponding to its relatively lower position in the overall expression ranking (indicating higher absolute expression). Conversely, *W. pigra* showed the lowest rank mean (6.6), reflecting its higher ranking position (denoting lower absolute expression) (Table 3). Mann–Whitney U test analysis demonstrated statistically significant differences in LDTI expression between all pairwise species comparisons (*p* < 0.05).

### 3.4. Recombinant Protein Expression

**Figure 2:**
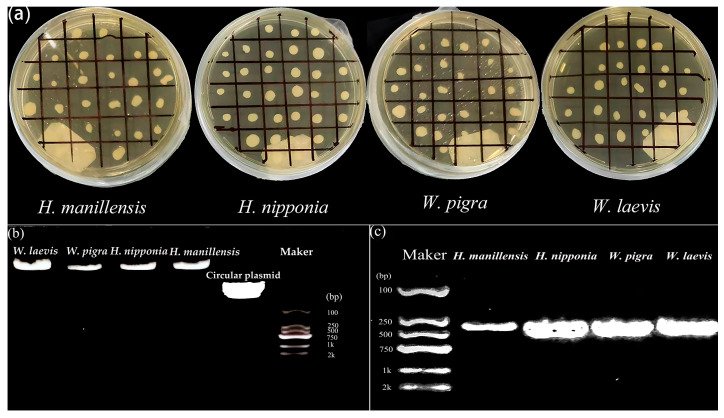
Verification of LDTI recombinant strain construction and expression in *Pichia pastoris*. (**a**) Transformant colonies selected on YEPD plates; (**b**) agarose gel electrophoresis (linearization of plasmid DNA); (**c**) agarose gel electrophoresis (translocation of the gene of interest).

The results demonstrated that chemically transformed *P. pastoris* colonies successfully grew on YEPD medium containing Geneticin, and two agarose gel electrophoresis analyses both revealed the corresponding bands, thereby indicating both successful linearization of the circular plasmid DNA and successful genomic integration (Figure 2).

### 3.5. Anticoagulation of LDTI

To investigate the anticoagulant effects of the LDTI protein at varying concentrations, this study conducted comprehensive analyses from both qualitative and quantitative perspectives. In the qualitative experiments, testing with the target protein at baseline concentration resulted in ACT values exceeding 30 min (beyond the instrument’s measurable range) for all samples, which was significantly higher than that of the control group, demonstrating a substantial prolongation of blood coagulation time (Table 4).

**Figure 3:**
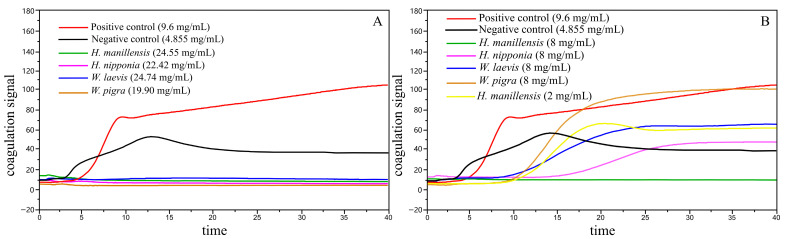
(**A**) Coagulation signal curves of the control group and four leech-derived LDTI proteins at baseline concentration. (**B**) Coagulation signal curves of four leech LDTI proteins at 8 mg/mL, *H. manillensis* LDTI at 2 mg/mL, and the control group. The *y*-axis indicates coagulation signal intensity, and the *x*-axis represents time (min). Positive control: PBS (phosphate-buffered saline) buffer; Negative control: empty vector protein.

In the qualitative experiments, no CR or PF values were detected in the target protein groups, indicating the absence of blood coagulation. This conclusion was further substantiated by coagulation signal curve analysis: the control group (red and black curve) exhibited a rapid increase in coagulation signal, reaching its peak at approximately 10 min, followed by a gradual decline, suggesting successful blood coagulation and stable clot formation. In contrast, the target protein groups (green, purple, blue, and brown curves) maintained consistently low coagulation signal levels throughout the observation period, with no significant changes, conclusively demonstrating the anticoagulant activity of the target protein (Figure 3A).

During the quantitative experimental phase, the target protein concentration was standardized to 8 mg/mL across three replicate experiments. The results revealed that, in terms of ACT values, *H. manillensis* samples were significantly higher than those of other leech species. In contrast, regarding CR and PF metrics, *H. manillensis* samples demonstrated lower values compared to *W. pigra* samples. This suggests that, at equivalent concentrations, the LDTI protein from *H. manillensis* exhibits superior anticoagulant activity relative to other leech species. Furthermore, compared to the initial concentration experimental group, the diluted target protein showed reduced anticoagulant activity but still maintained significant anticoagulant effects.

To further validate the anticoagulant potency of *H. manillensis* protein, its concentration was diluted to 2 mg/mL and subjected to triplicate experiments. The mean ACT, CR, and PF values measured were 551.5 s, 11.1 mm/min, and 0.7 AU, respectively. Comparative analysis of coagulation signal curves (Figure 3B) among the 8 mg/mL *H. manillensis* sample, the 2 mg/mL *H. manillensis* sample, and the control group demonstrated that, even at lower concentrations, *H. manillensis* protein maintains potent anticoagulant activity. This observation suggests robust stability and persistence in its anticoagulant efficacy.

## 4. Discussion

This study systematically investigated the genetic diversity and functional activity of the LDTI gene across four medicinal leech species. The results revealed significant interspecies differences in LDTI gene sequence variability, expression patterns, and the anticoagulant activity of its encoded proteins. Genetic diversity assessments at both the DNA and amino acid sequence levels showed marginally higher coefficients of variation in the non-hematophagous leeches *W. pigra* and *W. laevis* compared to the hematophagous species *H. manillensis* and *H. nipponia*. The evolutionary trajectory of leeches exhibits a unique phenomenon: shifts between hematophagous and non-hematophagous feeding ecologies. These dietary transitions may have influenced the molecular evolution of antithrombotic genes. Notably, in hematophagous leeches, anticoagulant genes—particularly LDTI, as a paradigmatic serine protease inhibitor—display conserved inhibitory specificity toward coagulation factor Xa (FXa). FXa occupies an upstream node in the coagulation cascade, where its inhibition produces potent anticoagulant effects through significant signal amplification [39]. Previous studies have confirmed FXa as the primary target for most antithrombotic genes in leeches [40,41]. Consequently, we posit that in hematophagous leeches, the functional conservation of LDTI genes is critical for survival. In contrast, reduced dependence on LDTI-mediated anticoagulation in non-hematophagous lineages may facilitate the accumulation of neutral mutations, potentially driving functional divergence or degradation.

Selection pressure analyses indicated that the LDTI gene family is predominantly subject to purifying selection at the genomic level. However, branch model analysis revealed a notable exception: *Hirudinaria manillensis* emerged as the only species exhibiting pervasive purifying selection across its LDTI locus, whereas orthologs in other studied species primarily evolved under neutral selection or weak positive selection signals. The genus Hirudinaria (e.g., *H. manillensis*, *H. javanica*, *H. bpling*, *H. thailandica*) [8,42] constitutes a monophyletic clade in which all known members are hematophagous. In contrast, the genera Hirudo and Whitmania are phylogenetically polyphyletic but fall under the same taxon. Consequently, both *H. nipponia* and species of Whitmania (e.g., *W. pigra*) exhibit significantly weaker hematophagous traits compared to the Hirudinaria taxon. Although *H. nipponia* is regarded as a typical hematophagous leech, numerous national and international studies have found that its antithrombin activity is much lower than that of *H. manillensis* [8]. These findings demonstrate that the evolutionary stability and obligate nature of hematophagy are key factors influencing the selection regimes acting on LDTI genes. This conclusion is further supported by branch-site model analyses: hematophagous leeches (e.g., *H. manillensis*) primarily undergo purifying and neutral selection, whereas non-hematophagous lineages (e.g., *W. pigra*) show patterns of positive selection—aligning closely with previous branch model results and functional activity comparisons.

Transcriptome-based analysis of relative expression levels revealed significant divergence in LDTI gene expression across leech species, with pronounced interspecific variability observed in all pairwise comparisons. Notably, hematophagous leeches exhibited substantially higher TPM values compared to their non-hematophagous counterparts. Specifically, *H. manillensis* (hematophagous) displayed peak expression, while *W. pigra* (non-hematophagous) showed minimal expression, forming a distinct expression gradient: *H. manillensis* > *H. nipponia* > *W. laevis* > *W. pigra*. Integrating sequence variation and selection pressure patterns, this divergence likely arises from distinct evolutionary adaptations: In *H. manillensis*, long-term specialization on mammalian hosts has optimized LDTI for efficient anticoagulation. Purifying selection preserves critical functional domains from deleterious mutations, while high-expression strategies ensure rapid inhibition of host coagulation—essential for sustained blood-feeding. In contrast, as a non-hematophagous species, *W. pigra* experiences positive selection that drives genetic variation to accommodate diverse feeding ecologies, with reduced expression minimizing energetic costs.

In vitro anticoagulation assay results further support this inference. Recombinant LDTI proteins from all four leech species exhibited potent anticoagulant activity at the initial concentration (ACT > 2369 s), confirming the effective blockade of the coagulation cascade. Even when diluted to 8 mg/mL, anticoagulant activity remained significantly higher than the control group. Notably, *H. manillensis* recombinant LDTI displayed the strongest activity: at an 8 mg/mL concentration, it maintained superior activity (ACT = 2369 s), significantly surpassing the activity of other species. After further dilution to 2 mg/mL, its ACT value (551.5 s) still substantially exceeded normal blood coagulation time (typically <200 s), while extremely low CR and PF values indicated that *H. manillensis* recombinant protein retains high-efficiency, stable anticoagulation at low concentrations, demonstrating the strongest anticoagulant activity among the species studied. In contrast, *W. pigra* and *W. laevis* proteins exhibited lower activity, consistent with their gene expression patterns. These results validate the functional activity of LDTI genes and suggest that anticoagulant proteins from hematophagous leeches have greater potential for pharmaceutical applications.

Despite a 2000-year history of medicinal use in traditional Chinese medicine (TCM), leeches have increasingly been marginalized in modern medicine. This shift is largely attributed to perceived limitations of TCM, such as the relatively slow onset of action, complex mechanisms involving multiple components, and the lack of standardized quantitative criteria. In contrast, Western pharmaceuticals have gained prominence due to their rapid onset, well-defined mechanisms of action, and precise molecular targets. However, current Western anticoagulant agents (e.g., rivaroxaban, heparin, warfarin) are associated with inherent bleeding risks [43]. Even for drugs developed from leech-derived hirudin and its derivatives, such as hirulogs (e.g., bivalirudin, desirudin), which demonstrate improved safety profiles compared to traditional anticoagulants, they still cannot fully avoid bleeding complications due to their direct targeting of thrombin [44,45]. Based on previous studies, LDTI targets coagulation FXa, which is upstream of thrombin. This distinctive mechanism of action provides a significant safety advantage [46]. Consequently, LDTI may serve as an ideal alternative to hirudin-based therapeutics, offering a superior option for anticoagulation therapy.

It should be noted that current research on leech-derived anticoagulant proteins remains in the early exploratory stages. In particular, obtaining highly active monomeric forms through in vitro expression systems (e.g., prokaryotic/eukaryotic expression) presents significant challenges. Consequently, extracting natural anticoagulant proteins from leech medicinal materials or utilizing leech materials directly in their traditional medicinal form represents a relatively feasible approach for acquiring these bioactive compounds under current technological constraints. However, these strategies are critically dependent on a stable and sustainable supply of leech resources. At present, domestic pharmaceutical R&D for leech-derived anticoagulants primarily relies on *H. nipponia* and *W. pigra* as raw materials. However, *H. nipponia* populations have undergone drastic declines due to intensive agrochemical use, progressive habitat loss, and overexploitation [47,48]. This study reveals that although *H. nipponia* and *W. pigra* are two species included in the Chinese Pharmacopoeia, *H. manillensis* is only listed in regional pharmacopoeias. However, the LDTI gene of *H. manillensis* exhibits higher sequence conservation, elevated expression levels, and enhanced anticoagulant activity—coupled with its broad distribution across Southeast Asia and suitability for large-scale aquaculture. These advantages position *H. manillensis* as an ideal alternative raw material to replace the endangered *H. nipponia* and suggest a novel direction for R&D in antithrombotic drug development. Given these compelling benefits, we strongly recommend its expedited inclusion in the Chinese Pharmacopoeia as one of the most promising antithrombotic medicinal resources.

## 5. Conclusions

This study presents the first systematic comparison of genetic diversity and functional activity of the LDTI gene between hematophagous and non-hematophagous leeches, revealing interspecific variations in sequence polymorphism, gene expression, and protein function. These findings not only enhance the understanding of leech-derived antithrombotic LDTI genes but also identify the recombinant LDTI protein from *H. manillensis*—with its high expression yield and potent bioactivity—as a promising candidate for future pharmaceutical development. Future research will expand sample sizes to include a broader range of leech species and antithrombotic gene families, comprehensively assessing genetic diversity to establish a solid theoretical foundation for antithrombotic drug discovery and support the sustainable exploitation of medicinal leech resources.

## Figures and Tables

**Table 1:** Intraspecific genetic variants of LDTI genes and their encoded proteins.

Species	Length (bp)	Coding Sequence			Protein Sequence		
		VS	HN	WD	VS	HN	WD
*Hirudinaria manillensis*	360	3	3	0.00556	1	2	0.00901
*Hirudo nipponia*	348	4	5	0.00533	1	2	0.00833
*Whitmania pigra*	348	6	8	0.00665	4	4	0.01881
*Whitmania laevis*	360	8	6	0.01007	1	2	0.00862
Total	—	21	22	—	7	10	—

Note: VS, number of variable sites; HN, number of haplotypes; WD, Watterson’s Theta diversity.

**Table 2:** Molecular evolutionary analysis of LDTI genes under different selection models.

Gene	Foreground of Branch Model	Foreground of Branch-Site Model		
		Purifying Selection	Neutral Selection	Positive Selection
*LDTI_Hman*	*ω* = 0.47	*P* = 45%, *ω* = 0.02	*P* = 55%, *ω* = 1	—
*LDTI_Hnip*	*ω* = 1.11	*P* = 45%, *ω* = 0.02	*P* = 55%, *ω* = 1	—
*LDTI_Wpig*	*ω* = 1.02	*P* = 17%, *ω* = 0.01	*P* = 20%, *ω* = 1	*P* = 63%, *ω* = 1.56
*LDTI_Wlae*	*ω* = 1.28	*P* = 17%, *ω* = 0.01	*P* = 20%, *ω* = 1	*P* = 63%, *ω* = 1.56

Note: *ω*, *ω* = *dN/dS*; *P*, Percentage.

**Table 3:** Statistical summary of relative expression levels of LDTI genes across four leech species.

Species	N	Mean ± SD	Median	Rank Mean
*H. manillensis*	10	2942.07 ± 1593.12 ^a^	2850.8	34.9
*H. nipponia*	10	860.28 ± 485.39 ^b^	735.97	24.9
*W. pigra*	10	32.03 ± 48.57 ^d^	12.04	6.6
*W. laevis*	10	321.61 ± 414.62 ^c^	230.27	15.6

Note: Different superscript letters (a–d) show statistically significant difference between TPM values of different genes.

**Table 4:** Anticoagulant activities (mean ± SD) of recombinant LDTI at different concentrations.

**Treat**	**ACT (s)**	**CR (mm/min)**	**PF (AU)**
Positive control (9.6 mg/mL)	230.7 ± 14.5	24.4 ± 3.0	2.7 ± 0.3
Negative control (4.855 mg/mL)	238.7 ± 57.8	11.0 ± 1.7	0.8 ± 0.7
*H. manillensis* (24.55 mg/mL)	—	—	—
*H. nipponia* (22.42 mg/mL)	—	—	—
*W. pigra* (19.90 mg/mL)	—	—	—
*W. laevis* (24.74 mg/mL)	—	—	—
*H. manillensis* (8 mg/mL)	2369.0 ± 0.0 ^a^	—	—
*H. nipponia* (8 mg/mL)	1075.3 ± 235.9 ^b^	3.6 ± 1.6	0.3 ± 0.0
*W. pigra* (8 mg/mL)	531.0 ± 69.9 ^c^	15.0 ± 2.6	1.1 ± 0.4
*W. laevis* (8 mg/mL)	553.0 ± 20.1 ^c^	7.2 ± 1.0	0.3 ± 0.1
*H. manillensis* (2 mg/mL)	652.0 ± 17.4	11.2 ± 0.9	0.4 ± 0.1

Note: ACT, activated clotting time; CR, clot rate; PF, platelet function; “—” indicates complete inhibition of coagulation, with no detectable clotting signal. Positive control: PBS (phosphate-buffered saline) buffer; negative control: empty vector protein. Different superscript letters (a–c) show statistically significant difference between ACT values of different genes.

## Data Availability

Data are contained within the article and Supplementary Files.

## Supplementary Materials

The following supporting information can be downloaded at https://www.mdpi.com/article/10.3390/biology14091247/s1. File S1: Coding and amino acid sequences of LDTI from *H. manillensis*, *H. nipponia*, *H. tianjinensis*, *H. medicinalis*, *W. pigra*, *W. laevis*, and *W. acranulata*; File S2: LDTI coding sequences from 10 samples of *H. manillensis*, *H. nipponia*, *W. pigra*, and *W. laevis*.

## Abbreviations

The following abbreviations are used in this manuscript:

TCM	traditional Chinese medicine
BMGY	buffered glycerol-complex medium with yeast extract
BMMY	buffered methanol-complex medium with yeast extract
VS	number of variable sites
HN	number of haplotypes
WD	Watterson’s Theta diversity
TPM	transcripts per million
ACT	activated clotting time
CR	clot rate
PF	platelet function
PBS	phosphate-buffered saline
